# Fully automated dried blood spot sample preparation enables the detection of lower molecular mass peptide and non-peptide doping agents by means of LC-HRMS

**DOI:** 10.1007/s00216-020-02634-4

**Published:** 2020-04-16

**Authors:** Tobias Lange, Andreas Thomas, Katja Walpurgis, Mario Thevis

**Affiliations:** 1 Center for Preventive Doping Research – Institute of Biochemistry, German Sport University Cologne, Am Sportpark Müngersdorf 6, 50933 Cologne, Germany; 2 European Monitoring Center for Emerging Doping Agents (EuMoCEDA), Cologne/Bonn, Germany

**Keywords:** Dried blood spots (DBS), Hematocrit (Hct), Growth hormone–releasing peptides (GHRP), TAP blood collection device, Doping, Sport

## Abstract

The added value of dried blood spot (DBS) samples complementing the information obtained from commonly routine doping control matrices is continuously increasing in sports drug testing. In this project, a robotic-assisted non-destructive hematocrit measurement from dried blood spots by near-infrared spectroscopy followed by a fully automated sample preparation including strong cation exchange solid-phase extraction and evaporation enabled the detection of 46 lower molecular mass (< 2 kDa) peptide and non-peptide drugs and drug candidates by means of LC-HRMS. The target analytes included, amongst others, agonists of the gonadotropin-releasing hormone receptor, the ghrelin receptor, the human growth hormone receptor, and the antidiuretic hormone receptor. Furthermore, several glycine derivatives of growth hormone–releasing peptides (GHRPs), arguably designed to undermine current anti-doping testing approaches, were implemented to the presented detection method. The initial testing assay was validated according to the World Anti-Doping Agency guidelines with estimated LODs between 0.5 and 20 ng/mL. As a proof of concept, authentic post-administration specimens containing GHRP-2 and GHRP-6 were successfully analyzed. Furthermore, DBS obtained from a sampling device operating with microneedles for blood collection from the upper arm were analyzed and the matrix was cross-validated for selected parameters. The introduction of the hematocrit measurement method can be of great value for doping analysis as it allows for quantitative DBS applications by managing the well-recognized “hematocrit effect.”

**Electronic supplementary material:**

The online version of this article (10.1007/s00216-020-02634-4) contains supplementary material, which is available to authorized users.

**Figure:**
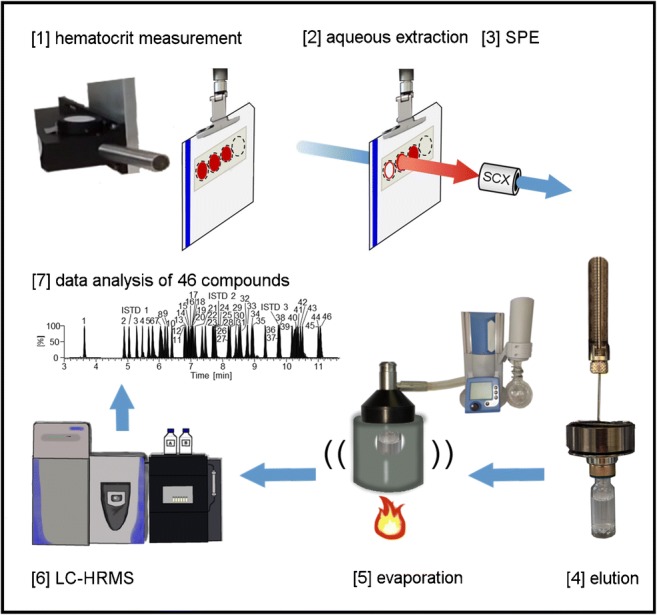
Graphical abstract

## Introduction

The use of prohibited peptidic drugs and non-peptide mimetics of lower molecular mass (< 2 kDa) to illegally increase performance in professional sports has been in the focus of preventive doping research for almost a decade. In 2010, the first LC-MS detection method for growth hormone–releasing peptide-2 (GHRP-2) in human urine was described [1] following the identification of the peptide in a nutritional supplement [2]. Subsequently, detection methods were developed and the analytical spectrum was continuously expanded. While initially mainly SPE extracts from urine samples were used for the detection of lower molecular mass peptides by anti-doping laboratories [1, 3–9], more recent approaches include a total of 21–36 target peptides or their metabolites which can be directly detected from urine by LC-HRMS (“dilute-and-inject”) [10–12]. The growing list of analytes comprises agonists of the ghrelin receptor (e.g. the GHRPs), the gonadotropin-releasing hormone (GnRH) receptor, the human growth hormone (hGh) receptor, and the antidiuretic hormone (ADH) receptor. As they act on different biological axes, their variety of performance-enhancing effects range from fat loss, bone formation, muscle and blood vessel growth to the masking of prohibited substances [4, 13]. Since the World Anti-Doping Agency (WADA) listed the GHRFs (growth hormone–releasing factors) including growth hormone secretagogues (GHS) and GHRPs in 2013 under section S2 “peptide hormones, growth factors, related substances, and mimetics”, several adverse analytical findings (AAFs) were reported, mostly from strength sports, which can be attributed to the anabolic effects of these drugs. From 2016 to 2017, the number of GHRF testing was increased by 17% [14]. Recently, glycine-modified analogues of GHRP-2, GHRP-6, and ipamorelin were identified in seized material [15–17]. All of these compounds are classified as non-threshold substances and are prohibited by WADA at all times [18] (in- and out-of-competition).

Urine has been the preferred matrix for the detection of these lower molecular mass peptides in routine doping controls as most analytes demonstrated sufficient stability in urine. Pharmacokinetic studies have demonstrated rapid elimination rates for GHRP-2 [19] and GHRP-6 [20] from blood with a biological half-life of 2.5 ± 1.1 h for GHRP-6. After intravenous (i.v.) administration of GHRP-2, detection times appeared shorter in serum than in urine samples, but for GHRP-6, detection windows were found to be comparable in both matrices [21, 22]. Nevertheless, for urine analysis, knowledge about the metabolic fate of peptide drugs is desirable as the presence of metabolites alongside the intact and unmodified drug (candidate) was shown in the past [3, 9, 23]. For example, GHRP-1 and alexamorelin are rapidly degraded and are traceable in urine only with significantly inferior sensitivity when compared with their metabolites [11].

Dried blood spots (DBS) represent an alternative matrix, which was found to be minimally invasive, cost-efficient, and analyte-stabilizing. Furthermore, sample preparation and analysis were automatable [24, 25] with the prospect of effective high-throughput testing. However, the limited sample volume of 10–20 μL blood, obtained, e.g., from the fingertip, and the highly complex matrix including hemolyzed blood cells and a high content of soluble and insoluble proteins pose a challenging task for sports drug testing laboratories. This suggests the use of modern chromatographic-mass spectrometric instrumentation, preferably in combination with an automated DBS sample preparation workflow, to enable testing for physiologically relevant concentration levels for these compounds without extensive manual sample preparation such as affinity enrichment. Another aspect to consider in DBS analysis is the “hematocrit (Hct) effect” [26, 27]. The influence of blood dispersal on the DBS filter paper was described to result in a Hct-dependent bias in quantitative assays, and the determination of Hct in DBS may contribute to overcome this limitation.

The aim of this study was to develop and optimize a fully automated DBS sample preparation as a multi-analyte initial testing approach for 46 lower molecular mass peptide and non-peptide agonists. The subsequent LC-HRMS detection method was validated according to WADA guidelines and reconstructed post-administration DBS samples containing GHRP-2 and GHRP-6 were successfully analyzed for proof-of-concept. Moreover, an upstream near-infrared (NIR) spectroscopic measurement was envisaged to support the non-destructive Hct determination before starting with the sample extraction as described by Oostendorp et al. [28]. In the context of anti-doping research, fully automated determination of small molecules from DBS was already achieved as, for example, for nicotine and adipoRon (a synthetic adiponectin receptor agonist) with LODs of 5 ng/mL [24, 25].

## Materials and methods

### Chemicals and materials

Ammonium hydroxide, acetonitrile, acetic acid, methanol, and MiniPax® absorbent packets were obtained from Merck (Darmstadt, Germany). Albumin solution 20% (v/v) was purchased from Carl Roth (Karlsruhe, Germany). Formic acid was bought from Thermo Fisher Scientific (Bremen, Germany), dimethyl sulfoxide (DMSO) was supplied by Alfa Aesar (Haverhill, MA, USA), and Whatman™ FTA® DMPK-C sample collection cards were obtained from GE Healthcare (Uppsala, Sweden). For blood collection from the upper arm, “TAP” microneedle-based devices were purchased from Seventh Sense Biosystems (Cambridge, MA, USA), and for blood collection from the finger, a Microlet 2 lancing device with lancets from Bayer AG (Leverkusen, Germany) was used. The GHRP-2 metabolite and d_3_-Ala-GHRP-2 metabolite (ISTD 2) were in-house synthesized as described elsewhere [3]. A total of 47 peptide and non-peptide compounds, including 45 analytes and 2 internal standards (ISTDs), were purchased from different suppliers: Auspep (Melbourne, Australia), Bachem (Bubendorf, Switzerland), BMFZ (Düsseldorf, Germany), Centic Biotec (Heidelberg, Germany), Genscript (Piscataway, NJ, USA), MedChem Express (Princeton, NJ, USA), Pepscan (Lelystad, Netherlands), Prospec (Rehovot, Israel), Sigma Aldrich (St. Louis, MO, USA), Sanofi (Paris, France) and Toronto Research Chemicals (North York, ON, Canada). The reference material had a specified purity between 90 and 99% and a specified peptide content between 60 and 94% (see Electronic Supplementary Material (ESM) Table S1).

### Standard solutions

Standard and ISTD stock solutions of the peptides were prepared in Milli-Q water with 10% acetonitrile, 2% acetic acid, and 0.5% albumin (v/v) in LoBind tubes. For peptides that were hardly soluble, more acetic acid was added (GHRP-1 met.: 6%, Gly-GHRP-4: 3%, Gly-GHRP-5: 6%). The stock solutions had concentrations between 0.5 and 1 mg/mL and were stored at − 20 °C. A standard stock mix of all compounds was prepared by diluting the stock solutions to 10 μg/mL (− 20 °C). Working solutions of the analytes (25–1000 ng/mL) and ISTD (100 ng/mL) were freshly prepared with the solvent mixture used to prepare the aforementioned stock solutions.

### DBS sampling methods

EDTA-stabilized blood samples from healthy volunteers were used as a matrix for the preparation of DBS during method development, optimization, and validation. The blood was fortified with the desired concentration of the peptide standard and mixed briefly before spotting 20 μL onto a DBS card. To demonstrate specificity and identification capability, capillary whole blood from the fingertip (by micro lancet) or upper arm (by “TAP” blood collection device) was obtained from five female and five male volunteers. After pricking the fingertip, the first drop of blood was wiped off and then 20 μL were taken with a pipette and placed onto a DBS card. The microneedle-based “TAP” blood collection device combines capillary action and vacuum extraction to collect capillary blood from the upper arm into a Li-heparin coated chamber (100 μL) [29]. After several minutes, the completion of the blood collection is displayed by a blood indicator window. A pipette was used to transfer 20 μL from the device onto the filter paper. Unless otherwise stated, DBS cards were dried for 2 h at room temperature (RT) and then stored overnight at 4 °C in plastic bags with desiccant.

### Post-administration samples

From a previous application study, EDTA plasma samples were available from a male subject (59 years, 78 kg) [30]. Here, a single injection containing 666 μg of GHRP-2 and 200 μg of GHRP-6 (Hallandale Pharmacy, FL, USA) was administered subcutaneously. Serum samples were collected after 30, 90, and 270 min and stored at − 20 °C. For the preparation of artificial DBS specimens, the serum was mixed carefully with fresh blood cells (obtained from EDTA-stabilized venous blood) up to a Hct of 40%.

### Fully automated measurement of Hct and sample preparation

The determination of the Hct was realized using a NIRFlex N-500 spectrometer equipped with a fiber optics solids cell from Büchi Labortechnik AG (Essen, Germany) connected to an automated DBS sample preparation system from Gerstel (Mülheim an der Ruhr, Germany). Before initiating sequenced measurements, an internal reference spectrum was recorded by an internal calibration based on a NIR model designed by Oostendorp et al. [28] using 261 patient DBS samples (EDTA full blood) with different Hct, age, and sex. After every 10 measurements, a white reference cap was placed manually in front of the fiber optic probe in order to perform a white balance (external calibration). The calibrations were performed according to the manufacturer’s advice. DBS cards were automatically moved in front of the optic probe tip in order to non-destructively measure the Hct within 2–3 s. Robotic-assisted sample preparation was then accomplished by a dual-head multi-purpose sampler (MPS) interfaced with a DBS autosampler (DBSA), a solid-phase extraction (SPE) module loaded with strong cation exchange (SCX) polymer cartridges, a multi-position evaporation station (mVAP), and a high-pressure dispenser pump (HPD). The devices were controlled by the Gerstel Maestro 1 software (version 1.4.49.8) and NIRWare (version 1.5.3000).

### LC-HRMS/MS

LC-HRMS/MS analysis was accomplished using a Vanquish UHPLC system coupled to a Q Exactive™ HF-X Hybrid Quadrupole-Orbitrap™ mass spectrometer, both from Thermo Fisher Scientific (Bremen, Germany) with nitrogen as source/collision gas (CMC, Eschborn, Germany). A Poroshell 120 EC-C8 analytical column, 3.0 × 50 mm, 2.7 μm PS from Agilent Technologies (Santa Clara, CA, USA) separated the analytes chromatographically with 0.1% formic acid as solvent A and acetonitrile, 0.1% formic acid, and 1% DMSO as solvent B, with a flow rate of 350 μL/min. After the injection of 10–20 μL of the sample into the instrument, the chromatographic run with an overall runtime of 15 min was as follows: 1–40% B over 10 min, 40–90% B in 0.5 min, 90% B over 1.5 min, and 1% B for 3 min. The temperature of the sampler was set to 10 °C, the column compartment to 30 °C and the transfer capillary to 320 °C. The ion source was operated in positive mode with an ionization voltage of 3.3 kV. The MS analysis comprised alternating full scan MS experiments with a scan range from m/z 300 to 1500 and targeted-SIM (t-SIM)/dd-MS^2^ experiments with an inclusion list of 53 ions. The resolution (FWHM at m/z = 200) was set to 60,000 (full MS), 45,000 (t-SIM), and 15,000 (dd-MS^2^), automatic gain control target value to 3e6 (full MS), 2e5 (t-SIM), and 5e5 (dd-MS^2^), and maximum ion injection time to 200 ms (full MS), 25 ms (t-SIM), and 50 ms (dd-MS^2^), respectively. The t-SIM experiments were acquired with a retention time window of ± 0.5 min around the expected elution time of each analyte, with an isolation window of 3.0 m/z, and an offset of 1.0 m/z. A total of five scan events with a maximum number of five multiplexed ions were acquired before the next full MS started. The normalized collision energy was 35%. The instrument was operated by Thermo Scientific Xcalibur, version 4.1.31.9.

The t-SIM experiments were used to identify the substances by their precursor ions. Full scan MS experiments were acquired alternately with t-SIM/dd-MS^2^ experiments; thus enabling a retrospective data analysis for the detection of formerly unknown substances and metabolites by their precursor ions. The dd-MS^2^ data provided additional information to confirm the selected ion signals of the inclusion list However, unknown substances cannot be additionally identified via MS/MS within this method.

### Method validation of the initial testing procedure

The robotic-assisted DBS sample preparation with subsequent LC-HRMS detection for 46 analytes (Table 1) was validated according to WADA guidelines for the validation of initial testing procedures (ITPs) for non-threshold substances [31]. Each analyte was identified by t-SIM experiment at its retention time using two signals, referred to as target ion and confirming ion. The signals are isotopes of the respective precursor ions of the dominant charge state. Gly-GHRP-2 was an exception, as isotope signals of different charge states 1+ and 2+ were used for identification. Five male and five female volunteers were chosen to demonstrate specificity and identification capability. For the ‘identification capability’, the blood spots prepared from capillary finger or capillary upper arm blood were allowed to dry on the filter paper for 30 min before 4 μL of a 100 ng/mL standard mix was added onto the middle of the spot to obtain a concentration of 20 ng/mL. Varying Hct values (24–44%) from different individuals of these DBS samples were used to prove the assay’s robustness towards extractability-related issues. Since different limits of detection (LODs) were expected for the individual analytes, six sample replicates at different concentrations (0.5, 1, 2, 5, 10 and 20 ng/mL) were prepared. The precision was estimated using six sample replicates each at 20 ng/mL, 50 ng/mL, and 100 ng/mL and the coefficient of variation (CV) of the ISTD-normalized peak areas was calculated. In order to study linearity, a series of standards within the working range with 2, 5, 10, 20, 50, and 100 ng/mL was prepared and the ISTD-normalized peak areas were analyzed assuming a linear (1st order) regression. The analyte concentrations of the DBS for the validation parameters “LOD,” “linearity,” “precision,” and “carryover” were prepared by carefully mixing venous EDTA-blood with an appropriate amount of the standard mix (volume ≤ 5% of the total volume) before spotting onto the DBS card. The recovery was estimated by comparing six samples containing 20 ng/mL of the standard mix (pre-extraction) and six blank samples that were fortified with 20 ng/mL of all target analytes after the evaporation (post-extraction). The pre-extraction DBS samples were prepared by adding 4 μL of a 100 ng/mL standard mix onto an already dried 20 μL EDTA-blood spot (in the same way as for ‘identification capability’). Analytes were thus located in the center of the spot, which allowed their complete extraction by means of the 6-mm clamp. Prior to LC-MS analysis, pre-extraction samples were fortified with 4 μL ddH_2_0 and post-extraction samples were fortified with 4 μL of a 100 ng/mL standard mix and mixed briefly. Three blank samples and three neat samples each fortified with 20 ng/mL immediately before the LC-MS analysis were analyzed to determine absolute matrix effects. The stability of the analytes on the DBS cards as well as the stability of the Hct values was investigated with respect to different storage times (1, 2, 3, 7, 14, 21 days) and temperatures (− 20 °C, 4 °C, 20 °C). Two DBS sample replicates were prepared for each storage condition. LC-MS carryover was determined by analyzing a negative control sample (same matrix) immediately after a sample containing a high analyte concentration of 100 ng/mL. To further study the DBSA-SPE carryover, a blank sample was extracted after this sample. For the specificity and identification capability, a cross-validation for DBS obtained from the upper arm (“TAP” device) from the same ten volunteers was realized.

**Table 1:** LC-HRMS related characteristics and categories of the target compounds

Compound	Pre-dominant charge state	Target ion [m/z]	Confirming ion [m/z]	RT [min]	Category
Alarelin	2+	584.3065	584.8080	7.01	GnRH receptor agonist
Alexamorelin	2+	479.7560	480.2574	7.42	Ghrelin receptor agonist
Alexamorelin (3–6) met.	1+	623.2957	624.3030	10.35	Ghrelin receptor agonist
Anamorelin	1+	547.3391	548.3421	11.08	Ghrelin receptor agonist
AOD9604	2+	907.9375	908.4388	6.98	hGH receptor agonist
AOD9604 (7–16) met.	2+	521.7077	522.2092	5.27	hGH receptor agonist
Buserelin	2+	620.3353	620.8367	8.50	GnRH receptor agonist
(d_3_)-Ala-GHRP-2 met. (ISTD)	1+	361.1948	362.1979	7.68	Ghrelin receptor agonist
(d_4_)-Ala-GHRP-4 (ISTD)	1+	612.3231	613.3262	9.54	Ghrelin receptor agonist
Deslorelin	2+	641.8276	642.3291	8.54	GnRH receptor agonist
Desmopressin	1+	1069.4342	1070.4370	7.04	ADH receptor agonist
Felypressin	2+	520.7257	521.2271	6.16	ADH receptor agonist
Fertirelin	2+	577.2987	577.8001	6.81	GnRH receptor agonist
GHRP-1	2+	478.2505	478.7520	7.65	Ghrelin receptor agonist
GHRP-1 (3–6) met.	1+	620.2883	621.2913	11.02	Ghrelin receptor agonist
GHRP-2	2+	409.7210	410.7240	8.88	Ghrelin receptor agonist
GHRP-2 (1–3) met.	1+	358.1761	359.1792	7.69	Ghrelin receptor agonist
GHRP-3	1+	655.4038	656.4067	6.03	Ghrelin receptor agonist
GHRP-4	1+	608.2980	609.3010	9.74	Ghrelin receptor agonist
GHRP-5	1+	771.3613	772.3643	10.27	Ghrelin receptor agonist
GHRP-6	2+	437.2296	437.7312	6.81	Ghrelin receptor agonist
GHRP-6 (2–5) met.	1+	609.2820	610.2850	10.19	Ghrelin receptor agonist
Gly-Alexamorelin	2+	508.2667	508.7681	7.08	Ghrelin receptor agonist
Gly-GHRP-1	2+	506.7612	507.2627	7.71	Ghrelin receptor agonist
Gly-GHRP-2	2+	438.7330	876.4592	8.93	Ghrelin receptor agonist
Gly-GHRP-3	1+	712.4253	713.4281	6.24	Ghrelin receptor agonist
Gly-GHRP-4	1+	665.3194	666.3224	9.77	Ghrelin receptor agonist
Gly-GHRP-5	1+	828.3828	829.3858	10.52	Ghrelin receptor agonist
Gly-GHRP-6	2+	465.7403	466.2417	6.91	Ghrelin receptor agonist
Gly-Hexarelin	2+	472.7481	473.2496	6.98	Ghrelin receptor agonist
Gly-Ipamorelin	2+	385.2108	385.7123	6.79	Ghrelin receptor agonist
Goserelin	2+	635.3280	635.8294	8.17	GnRH receptor agonist
Hexarelin	2+	444.2374	444.7388	6.94	Ghrelin receptor agonist
Hexarelin (1–3) met.	1+	427.2088	428.2117	4.87	Ghrelin receptor agonist
Histrelin	2+	662.3409	662.8423	6.92	GnRH receptor agonist
Ibutamoren	1+	529.2479	530.2505	10.38	Ghrelin receptor agonist
Ipamorelin	2+	356.7001	357.2016	5.77	Ghrelin receptor agonist
Ipamorelin (1–4) met.	1+	585.2820	586.2850	7.65	Ghrelin receptor agonist
Lecirelin (dalmarelin)	2+	605.3300	605.8314	8.40	GnRH receptor agonist
Leuprolide	2+	605.3300	605.8314	8.16	GnRH receptor agonist
Leuprolide (1–3) met.	1+	453.1881	454.1910	5.27	GnRH receptor agonist
LHRH	2+	591.7938	592.2953	6.39	GnRH receptor agonist
[Lys8]-Vasopressin (ISTD)	2+	528.7231	529.2248	5.02	ADH receptor agonist
Nafarelin	2+	661.8251	662.8279	9.36	GnRH receptor agonist
Nafarelin (5–10) met.	2+	401.2242	401.7257	8.75	GnRH receptor agonist
Peforelin	2+	630.2889	630.7903	5.47	GnRH receptor agonist
Tabimorelin	1+	529.3173	530.3205	10.31	Ghrelin receptor agonist
TB500	2+	445.2531	445.7546	3.61	Synthetic version of an active region of thymosin β_4_
Triptorelin	2+	656.3227	656.8241	8.21	GnRH receptor agonist

## Results and discussion

### Method development and optimization

The fully automated sample preparation was optimized with regard to the extraction agent, the employed extraction volume, the SPE purification, and the duration, temperature, and vacuum of the evaporation step. Due to the good water solubility of the analytes, an aqueous solution was used for the extraction from the DBS card. Different stationary phases (CN, C12, C8, C18, SCX, strong hydrophobic, general-purpose, and mixed-mode cation/anion exchange) were tested for solid-phase extraction and ion exchange cartridges, especially SCX, yielded the best results.

Briefly, a DBS was extracted with 1.5 mL ddH_2_O (4 mL/min, 100 °C) through a clamp with a diameter of 6 mm. Sixty microliters of the deuterated ISTD mix (100 ng/mL) were automatically added through a separate loop and the sample extract was loaded onto a pre-conditioned SCX SPE. The SCX cartridge was washed with 2% formic acid and analytes were eluted with 1.4 mL of 5% ammonium hydroxide in methanol into a glass vial. Then, the sample eluate was evaporated in the mVAP for 37 min at 50 °C and 250 rpm with ramping pressure from 200 to 60 mbar. Finally, the sample containing 100 μL could be transferred manually to the LC-HRMS system.

With a cold system start, the total time for a batch of 6 samples (maximum number of positions in the mVAP) was approximately 2 h. By programming a nesting of individual sample preparation steps, the total time of various batches could be significantly reduced. More details about the individual steps and information about volumes, flow velocities, pressure, temperature, and agitation can be found in the ESM.

### Method characterization and validation

As shown in Table 1, a total of 46 target analytes were included within this study, comprising “classical” peptide drugs such as ipamorelin (pentapeptide) or goserelin (decapeptide) and non-peptide drugs (mimetics) such as anamorelin as well as several potentially performance-enhancing Gly-derivatives of the GHRPs. Moreover, GHRP-1, which was stable in urine only as its metabolite [10], was successfully analyzed in DBS. Felypressin, a new vasoconstrictor related to vasopressin, was determined for the first time.

The lower molecular mass peptides < 2 kDa were observed to predominantly form doubly-charged molecules under the chosen conditions. However, as already described by others, DMSO as an additive in the LC solvent does not only improve the ionization efficiency [32] but also influences the charge state distribution of the peptides, shifting the equilibrium to the direction of lower charge states [33]. Therefore, a relatively large number of 17 singly charged molecules compared with 29 doubly charged species were utilized for an unambiguous identification (target ion) within this assay (t-SIM experiments).

The assay was characterized by a homogeneous chromatographic distribution of the analytes with most substances eluting between 5 and 11 min.

**Fig. 1:**
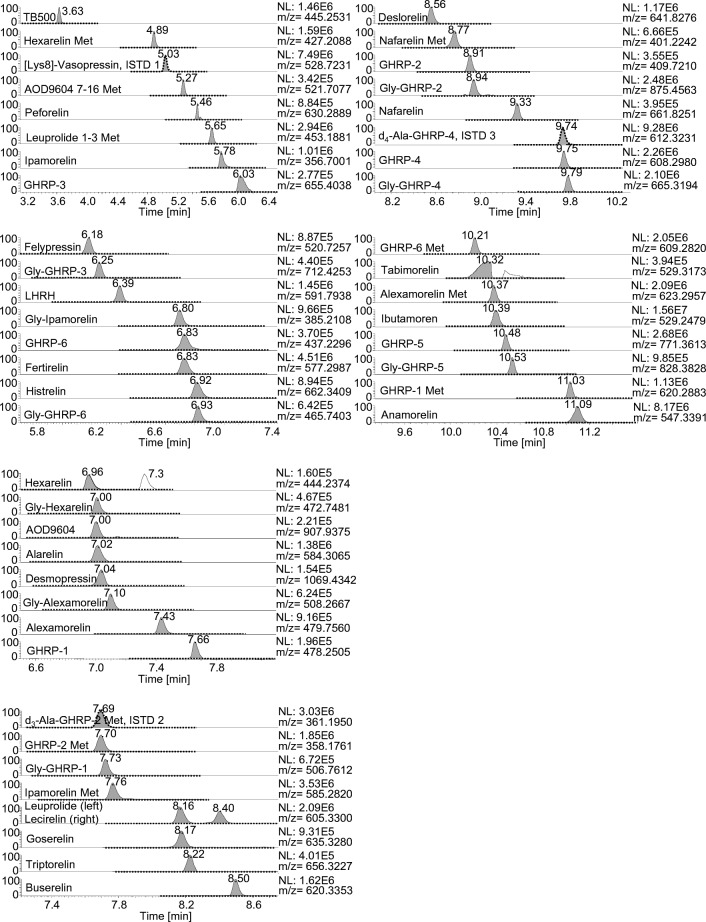
Extracted ion chromatograms (mass tolerance ± 5 ppm) of a sample from a female volunteer obtained by a finger prick. The sample was either analyzed as blank (dashed line) or with 20 ng/mL of a peptide mix containing all 46 target compounds. The rows of the 3 ISTDs are also shown at their respective retention time

The ITP was validated according to WADA’s international standard for laboratories 10.0 [31] (Table 2). As the analytes are non-threshold substances, the minimum criteria for LC-MS confirmation of the identity of analytes are applied to demonstrate the presence of a prohibited substance. The selectivity for the 46 substances was demonstrated by analyzing 10 blank samples collected from different individuals (male and female). As exemplarily shown in Fig. 1 (dashed line) and ESM Fig. S1, no interfering signals were detected in the blank samples (specificity). Subsequently, 10 samples from the same volunteers were fortified with 20 ng/mL of the standard mix and analyzed again (Fig. 1, solid line). Hereafter, all substances of interest could be unambiguously identified (identification capability). The LC conditions were very suitable for the efficient separation of the analytes with the only exception of tabimorelin (poor peak shape). In case of a suspicious finding, adapted LC conditions can be used for a “confirmation procedure” for tabimorelin. In the ion chromatogram at RT = 7.3 min, a second peak of hexarelin with an identical mass was observed that originated from alexamorelin metabolite (2–7)-NH_2_. Due to an intensive degradation of alexamorelin in blood as was shown by others [23, 34], several metabolites can be formed. This metabolite was not included for initial testing purposes; however, it may assist the confirmation of an alexamorelin finding. Following this, the alternative “TAP” sampling method for the upper arm was tested and cross-validated for these parameters. In the same way as for the blood collected from the fingertip, the assay’s selectivity was shown for all target analytes. Furthermore, the LOD was estimated by applying a signal-to-noise ratio > 3 for the individual target and confirming ions with the following results: 7 analytes at 0.5 ng/mL, 7 analytes at 1 ng/mL, 12 analytes at 2 ng/mL, 10 analytes at 5 ng/mL, 9 analytes at 10 ng/mL, and 1 analyte at 20 ng/mL.

**Table 2:** Main results of validation

Compound	LOD [ng/mL] (n = 6)	Linearity (R) LOD-100 ng/mL (*n* = 1)	Precision at 20 ng/mL [%] (*n* = 6)	Precision at 50 ng/mL [%] (*n* = 6)	Precision at 100 ng/mL [%] (*n* = 6)	Recovery at 20 ng/mL [%] (*n* = 6)	Carryover after 100 ng/mL [%] (*n* = 1)	Matrix effects [%] (*n* = 3)
Alarelin	5	0.9991	14.9	17.3	15.4	24.6	0.1	67
Alexamorelin	1	0.9967	16.8	17.5	7.8	9.9	8.5	113
Alexamorelin (3–6) met.	10	0.9979	31.2	35.3	21.9	13.0	0.1	49
Anamorelin	0.5	0.9998	15.2	16.4	5.2	33.7	0.7	48
AOD9604	10	0.9907	32.4	11.9	17.8	3.7	1.8	64
AOD9604 (7–16) met.	5	0.9888	37.7	22.4	11.9	10.5	0.6	54
Buserelin	5	0.9970	11.6	4.5	14.5	20.9	0	55
Deslorelin	5	0.9952	11.8	4.0	7.1	12.1	0.1	55
Desmopressin	10	0.9935	13.6	23.3	21.6	7.3	0	67
Felypressin	0.5	0.9990	14.7	6.3	3.6	25.5	0.7	86
Fertirelin	1	0.9993	14.5	14.5	18.5	22.1	0.1	69
GHRP-1	10	0.9919	21.8	17.4	12.7	11.2	3.6	142
GHRP-1 (3–6) met.	5	0.9991	18.4	53.4	8.9	14.9	0.1	48
GHRP-2	2	0.9991	13.3	5.2	6.8	11.2	0.1	55
GHRP-2 (1–3) met.	2	0.9998	17.5	7.8	5.3	25.1	0.1	76
GHRP-3	5	0.9973	19.5	2.7	27.8	27.4	0.1	79
GHRP-4	2	0.9996	15.7	14.7	2.6	28.2	0	53
GHRP-5	2	0.9964	16.8	14.6	4.8	22.4	0	33
GHRP-6	2	0.9975	7.9	15.2	25.6	41.6	9.1	96
GHRP-6 (2–5) met.	2	0.9982	24.7	31.6	10.6	19.8	0.1	52
Gly-Alexamorelin	5	0.9990	16.9	11.5	18.7	13.8	8.7	117
Gly-GHRP-1	2	0.9949	12.3	18.8	5.8	19.4	4.2	156
Gly-GHRP-2	10	0.9988	7.8	11.9	16.0	17.4	5.3	55
Gly-GHRP-3	5	0.9965	18.9	4.7	21.0	24.6	0.1	77
Gly-GHRP-4	1	0.9993	14.5	17.4	4.0	26.1	0	52
Gly-GHRP-5	5	0.9972	12.2	19.9	6.8	12.8	0.1	45
Gly-GHRP-6	2	0.9987	18.1	15.7	7.8	17.8	7.8	135
Gly-Hexarelin	2	0.9986	15.4	13.4	13.9	13.0	6.4	123
Gly-Ipamorelin	1	0.9984	10.0	12.6	9.9	69.6	7.8	125
Goserelin	2	0.9965	8.7	10.8	10.9	17.3	0.1	57
Hexarelin	10	0.9999	19.7	15.1	12.3	14.6	5.7	84
Hexarelin (1–3) met.	0.5	0.9958	25.7	14.9	5.6	24.4	6.9	51
Histrelin	5	0.9980	18.3	15.5	14.7	20.8	1.5	62
Ibutamoren	1	0.9999	24.9	15.5	13.2	46.3	0.4	46
Ipamorelin	0.5	0.9989	13.5	17.9	7.1	50.9	9.9	76
Ipamorelin (1–4) met.	0.5	0.9966	19.6	20.1	7.8	33.8	3.4	68
Lecirelin (dalmarelin)	0.5	0.9992	11.1	3.4	13.9	24.2	0	54
Leuprolide	0.5	0.9985	10.3	5.3	10.5	27.5	0	56
Leuprolide (1–3) met.	1	0.9981	23.1	16.7	11.5	23.6	0.1	75
LHRH	2	0.9992	14.4	4.7	24.0	21.4	0.2	70
Nafarelin	10	0.9862	13.8	4.0	8.3	8.0	0.2	49
Nafarelin (5–10) met.	2	0.9948	19.8	15.9	9.5	11.5	0.1	57
Peforelin	20	0.9985	26.6	12.8	14.0	34.9	0.5	61
Tabimorelin	10	0.9991	23.3	10.6	8.1	46.4	0	51
TB500	1	0.9996	22.2	12.8	6.7	19.5	1.6	54
Triptorelin	10	0.9961	15.5	3.5	7.3	14.0	0	56

For a more comprehensive assay characterization, additional parameters were determined as, for example, required for confirmation procedures. The individually variable Hct values of the DBS samples from a finger prick determined by the NIR spectrometer ranged between 24 and 40%. No impact on the LC-HRMS/MS identification of the analytes was observed, and the method’s robustness was demonstrated. Some articles reported on a Hct-dependent bias concerning quantitative bioanalysis using DBS, especially when a small punch of the DBS was excised [27]. Such phenomena were not observed in the present study, attributed to the fact that almost the entire spot was extracted using the 6 mm clamp, and the issue of nonhomogeneous analyte distribution described before was therefore negligible. However, within this assay, the robustness for post-fortified DBS samples (from blank samples with different Hct) was studied, since the smallest volume of non-coagulated capillary blood that could be obtained from the fingertip was not suitable for mixing with the standard before spotting onto the DBS card. Due to this way of DBS sample preparation, in which the analytes were pipetted onto the DBS card, the analytes were rather localized in the center of the spot. Thus, the impact of e.g. differential spreading of blood with different Hct was not evaluated here. The carryover after extracting a sample with a high concentration of 100 ng/mL was determined for both the DBSA and the LC-HRMS/MS system and was from 0 to 18.9% (data not shown) and from 0 to 9.9%, respectively. Despite the low probability of such highly concentrated doping control samples, it is recommended to rinse the DBSA system thoroughly on a regular basis to remove any potential residues of blood cell components and proteins. The total recovery of the method for the different analytes varied between 3.7 and 69.6%, and the matrix effects ranged from 33 to 156%. Values < 100% indicate ionization suppression effects and values > 100% indicate ionization enhancement effects caused by the sample matrix [35]. The linearity was determined from the LOD to 100 ng/mL or not lower than 2 ng/mL for analytes with LOD < 2 ng/mL and yielded coefficients of correlation *r* between 0.9862 and 0.9999. For the linear regression, slope and intercept were additionally specified (ESM Table S2). The precision was estimated for 6 replicates per analyte at 20 ng/mL, 50 ng/mL, and 100 ng/mL and was found to be below 25% for most substances. All analytes remained stable on the DBS card at all storage conditions over 3 weeks and were repeatedly identified at a concentration of 20 ng/mL for 2 replicates each. The variations of the observed ISTD-normalized peak areas were found to be within the range of variation of this method. A time-dependent degradation of the compounds could not be observed.

### Post-administration samples

**Fig. 2:**
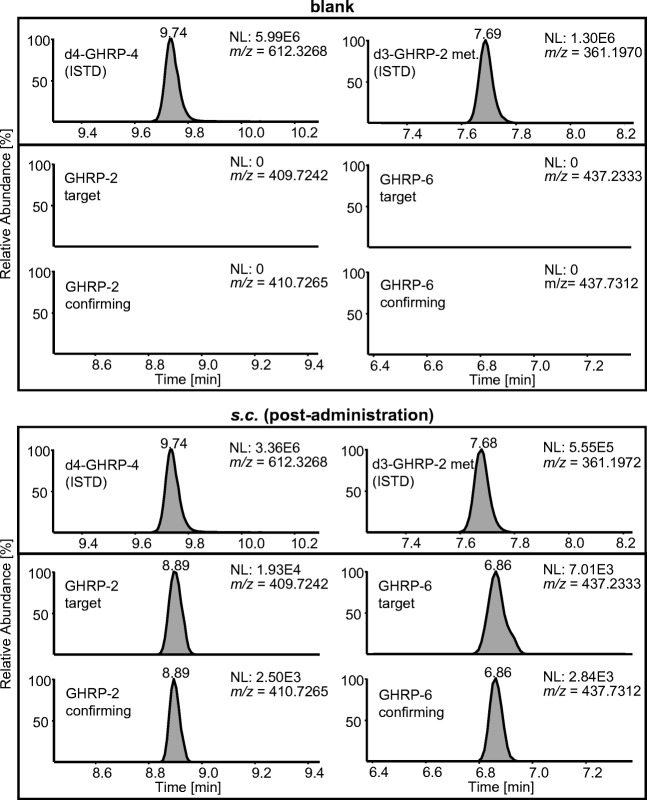
Extracted ion chromatograms (mass tolerance ±10 ppm) of a blank sample and a *s.c*. post-administration sample showing signals of GHRP-2 and GHRP-6

It is desirable to show that the substances cannot only be detected in fortified samples but also in authentic specimens, for example, collected in the course of administration studies. Since serum samples from a previous elimination study with GHRP-2 and GHRP-6 were available, DBS with a Hct = 40% were reconstructed. As shown in Fig. 2, both GHRP-2 and GHRP-6 could be unambiguously identified by their characteristic target and confirming ions in DBS generated from blood samples reconstructed with serum that was collected up to 90 min after application*.*

In DBS generated from blood reconstituted with serum that was collected 4.5 h post-administration, specific signals were still detectable but could not be confirmed by a second isotopic peak (confirming signal). A blank sample showed no interfering signals at the respective retention times. A standard calibration curve for GHRP-2 and GHRP-6 between 5 and 50 ng/mL was applied to estimate the concentration from the DBS samples, yielding levels between 2 and 10 ng/mL within the detection window (30–90 min). The results correspond with the concentrations of GHRP-2 and GHRP-6 after a single i.v. injection from other studies [19, 20]. It should be noted here that serum blood levels are expected to be slightly different from full blood levels because the analytes will most likely not be found at identical concentrations in red blood cells and serum. Nevertheless, the applicability of the testing procedure to post-administration samples could be successfully demonstrated.

### Hct determination by NIR spectroscopy

**Fig. 3:**
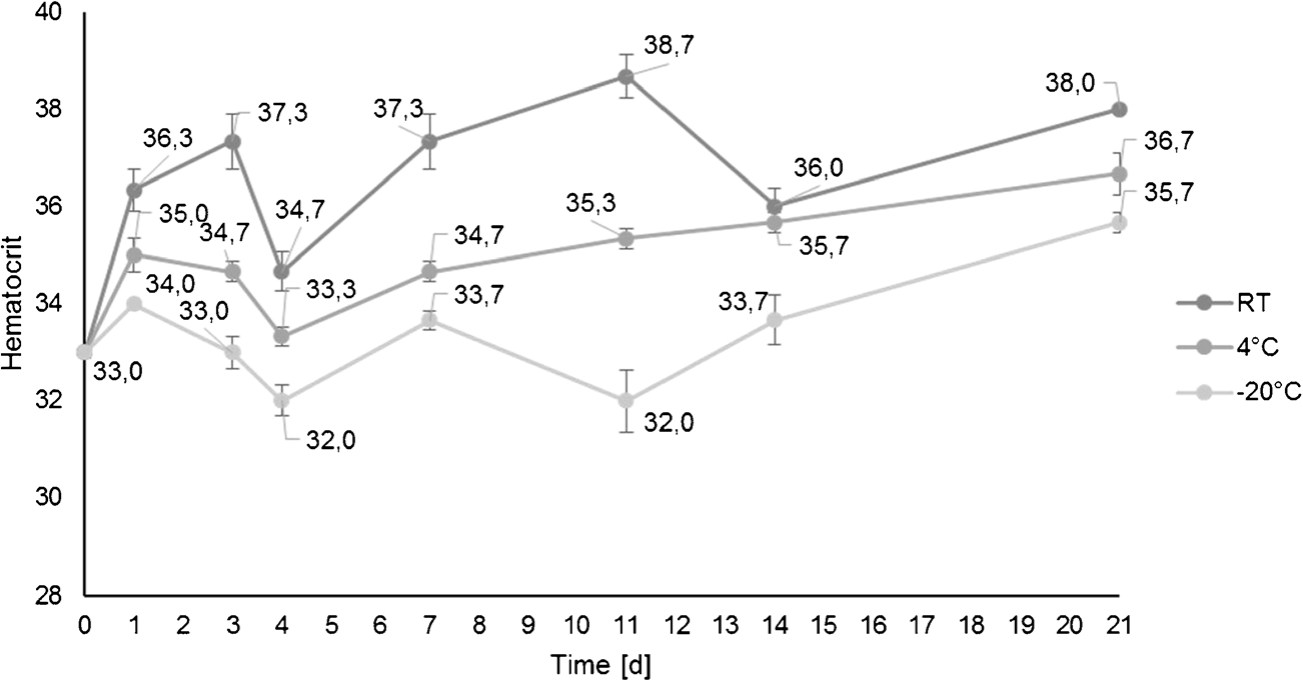
The influence of storage time and temperature on the Hct measurements from DBS by NIR spectroscopy was studied. Several DBS cards were stored over a period of 3 weeks at RT, 4 °C, and −  20 °C and samples were measured in triplicates (spots 1–3). The error bars result from the standard deviations of the respective experiments

The NIR spectroscope was used for non-destructive Hct measurements of DBS to obtain preliminary data in a pilot project only. A previously developed NIR model [28] was adopted to determine the Hct, and the influence of storage time and temperature on the Hct measurements was studied. For this purpose, EDTA-stabilized venous blood from one volunteer was utilized, and regular measurements in triplicate (spots 1–3) were performed over a period of 3 weeks while storing DBS cards in the dark at RT, 4 °C, and − 20 °C. DBS cards were dried for 6 h at RT and then stored under the conditions described above. A reference value of 38% measured by a Sysmex XN-1000 analyzer was determined on the first day after blood drawing. Regardless of the storage time, slight differences in a temperature-dependent manner in the range of 32.0–38.7% were observed. The DBS cards stored for at least 1 day at RT showed Hct values close to the reference value while cards stored at 4 °C and − 20 °C resulted in lower values as visualized in Fig. 3. In spite of sealing the cards in plastic bags with a desiccant, the differences in temperature or humidity might influence the molecular vibrations of the DBS matrix that are crucial for the NIR spectrum calculation. A time-dependent change in total hemoglobin was not assumed as others have already shown its stability in DBS [36]. A considerably slower drying or freezing of remaining moisture of the DBS matrix at reduced temperatures would be in accordance with this observation, suggesting a prolonged drying phase (1–2 days at RT followed by storage at RT with desiccant) for a reliable Hct measurement using the presented approach with DBS cards.

Since all measurements on the NIR spectroscope by Oostendorp et al. were based on DBS prepared from venous EDTA-stabilized blood [28], the applicability of the Hct calculation to capillary DBS collected by finger prick was investigated. Therefore, the Hct of different authentic DBS samples collected from ten volunteers was determined. DBS were sealed and dried for 2 days at RT. The measured Hct were found to be considerably lower than expected with a range from 24 to 40% (and an average of 31%), potentially resulting from different confounding factors. In some cases, finger blood collection was complicated by a slow blood flow, and even a slight squeezing of the finger can provoke exudate leaking into the collected blood causing a dilution of the sample. In addition, Hct values reportedly differ between body regions [37] and variation in capillary density, cutaneous blood content and red blood cell velocity must be taken into account [38]. The influence of anticoagulant (K2EDTA) on the NIR spectra might necessitate further investigations as well, suggesting more comprehensive studies in order to enable a holistic classification of Hct values obtained from NIR spectroscopy. As shown before, the different Hct values had no effect on the detection of the substances in this qualitative assay. However, the Hct is relevant in case of quantitative analyses regarding threshold substances determined from DBS, since the conversion from full blood into plasma concentrations seems to be decisive for the determination of concentration thresholds. A Hct-dependent correction factor could overcome this previous limitation when using DBS.

### DBS sampling methods

Both DBS sampling methods either from the arm or from the finger were successfully validated regarding selectivity. Compared with DBS collection from the finger prick, most volunteers described the arm device to be virtually painless and more comfortable to use. It is noteworthy that sample collection failure was reduced compared with the procedure with the finger lancet. The “TAP” devices could be leveraged to refine the method of blood collection.

## Conclusion

In sports drug testing, the demand for a higher sample throughput is continuously increasing. DBS sample collection may contribute to this development. The complementary matrix is mainly characterized by its cost efficiency (in terms of storage and shipping) and minimal invasiveness. In order to deter doping, a fully automated robotic DBS sample preparation with LC-HRMS detection was developed. The multi-analyte initial testing approach comprises 46 lower molecular mass peptide or non-peptide (mimetic) target analytes <2 kDa of different receptor agonist categories such as agonists that bind to ghrelin receptors, GnRH receptors, hGh receptors, and ADH receptors. Due to the discovery of glycine-modified analogues, the list of analytes was extended preventively with a series of nine glycine-modified peptides, mainly GHRPs. In addition, GHRP-1 that could not be detected in urine before [10], and felypressin, an ADH receptor agonist, were implemented for the first time in an anti-doping detection procedure. The vast majority of the drug candidates are still under development, in clinical trials or were discontinued. To the best of our knowledge, leuprolide, felypressin, LHRH, histrelin, desmopressin, GHRP-2, goserelin, triptorelin, buserelin, and nafarelin have obtained clinical approval. For veterinary use only, the application for marketing authorization was concerned for peforelin, alarelin, lecirelin, and deslorelin. Independent from the state of development, all these pharmaceuticals are available on the black market and pose a potential risk in relation to doping practices.

Despite the small DBS volume of 20 μL, sensitivities enabling the detection of an illicit use were achieved. Remarkably, more than 60% of the analytes could be detected below the WADA’s minimum required performance limit (MRPL) of 2 ng/mL for urine [39]. Until now, no MRPL specification is available for serum/plasma or DBS.

Furthermore, an upstream NIR spectroscope for non-destructive Hct measurement was implemented and the assay’s robustness in terms of extractability was demonstrated for different Hct values. This approach could contribute to a Hct-dependent correction and would support quantitative DBS applications in the future.

As a proof of concept, artificial DBS samples obtained from post-administration specimens containing GHRP-2 and GHRP-6 were successfully analyzed.

The automated DBS preparation of 6 samples lasted approximately 2 h, as long as the subsequent LC-HRMS analysis and was therefore ideally suited for a just-in-time workflow. The automation and the possibility of a programmable nesting of the preparation steps within the sequence would allow for an increased sample throughput compared with sophisticated manual sample preparation. Finally, if desired, the entire assay could be easily extended with new compounds.

## Electronic supplementary material

ESM 1 (PDF 625 kb)
