# Supplementary material for: Fully automated dried blood spot sample preparation enables the detection of lower molecular mass peptide and non-peptide doping agents by means of LC-HRMS
Source: Anal Bioanal Chem. 2020 Apr 16;412(15):3765–77. doi: 10.1007/s00216-020-02634-4 (PMC7220872; doi:10.1007/s00216-020-02634-4)
Supplement: Supplementary file 1 — (PDF 625 kb) [file 216_2020_2634_MOESM1_ESM.pdf]

Analytical and Bioanalytical Chemistry

Electronic Supplementary Material

Fully automated dried blood spot sample preparation enables the detection of lower molecular mass peptide and non-peptide doping agents by means of LC-HRMS

Tobias Lange, Andreas Thomas, Katja Walpurgis, Mario Thevis

DBS extraction

To ensure an efficient DBS extraction of the analytes, different organic-aqueous compositions were tested: 100%, 80%, 60%, 40%, 20%, and 0% acetonitrile. The addition of acetonitrile was found to reduce the co-elution of interfering proteins such as hemoglobin. At the same time, however, the amount of extracted analytes also decreased with increasing amounts of acetonitrile. Finally, an aqueous extraction (no organic component) was applied. Further purification of the DBS extract was achieved by a subsequent strong cation exchange (SCX) solid phase extraction (SPE).

Programming the automated DBS sample preparation

The protocol for the automated sample preparation was programmed as follows: First, a SCX SPE cartridge is loaded into position, conditioned with 1 mL methanol (3 mL/min) and washed with 1 mL water (3 mL/min). Second, the right arm of the MPS takes the first DBS card from the sample rack (maximum capacity: 40 cards) and inserts it into the DBSA. There, the position of the spot is determined by a camera recognition system and the card is moved in front of a 6 mm clamp, which now closes with 2900 N around the spot. Third, 60 µL of the deuterated ISTD mix (100 ng/mL) are automatically added through a separate loop before 1.5 mL aqueous flow extraction starts with 4 mL/min at 100°C. To avoid carry-over, the first 300 µL of the extract are discarded. For the same reason, the clamp now moves to an empty position on the DBS card and is rinsed with 1 mL water, 1 mL 80% acetonitrile, and 2 mL water. Meanwhile, the sample extract is loaded onto the SCX cartridge and is washed with 1 mL of 2% formic acid (3 mL/min). Fourth, the analytes are eluted with 1.4 mL of 5% ammonium hydroxide in methanol (2 mL/min) and transferred via the syringe of the left arm into a glass vial (10 mm vial penetration). Again, the first 50 µL of the eluate are discarded. The syringe is rinsed twice with 20% acetonitrile and water (fill/eject speed is 50 µL/s), before fifth, it is transported to the mVAP and there the eluate is evaporated by agitation (250 rpm) for 37 minutes at 50°C and ramping pressure from 200-60 mbar. The mVAP offers the simultaneous handling of a maximum of 6 samples and is therefore the limiting factor of the sample preparation workflow. Finally, the syringe is again rinsed twice and the sample containing 100 µL is now ready for LC-MS analysis.

The software allows a nesting of the preparation steps within the sequence. This means, for example, that 6 blood spots can be extracted while the robot is still evaporating the previous 6 samples. That results in a considerable reduction of total sample preparation time.

Supplementary figures

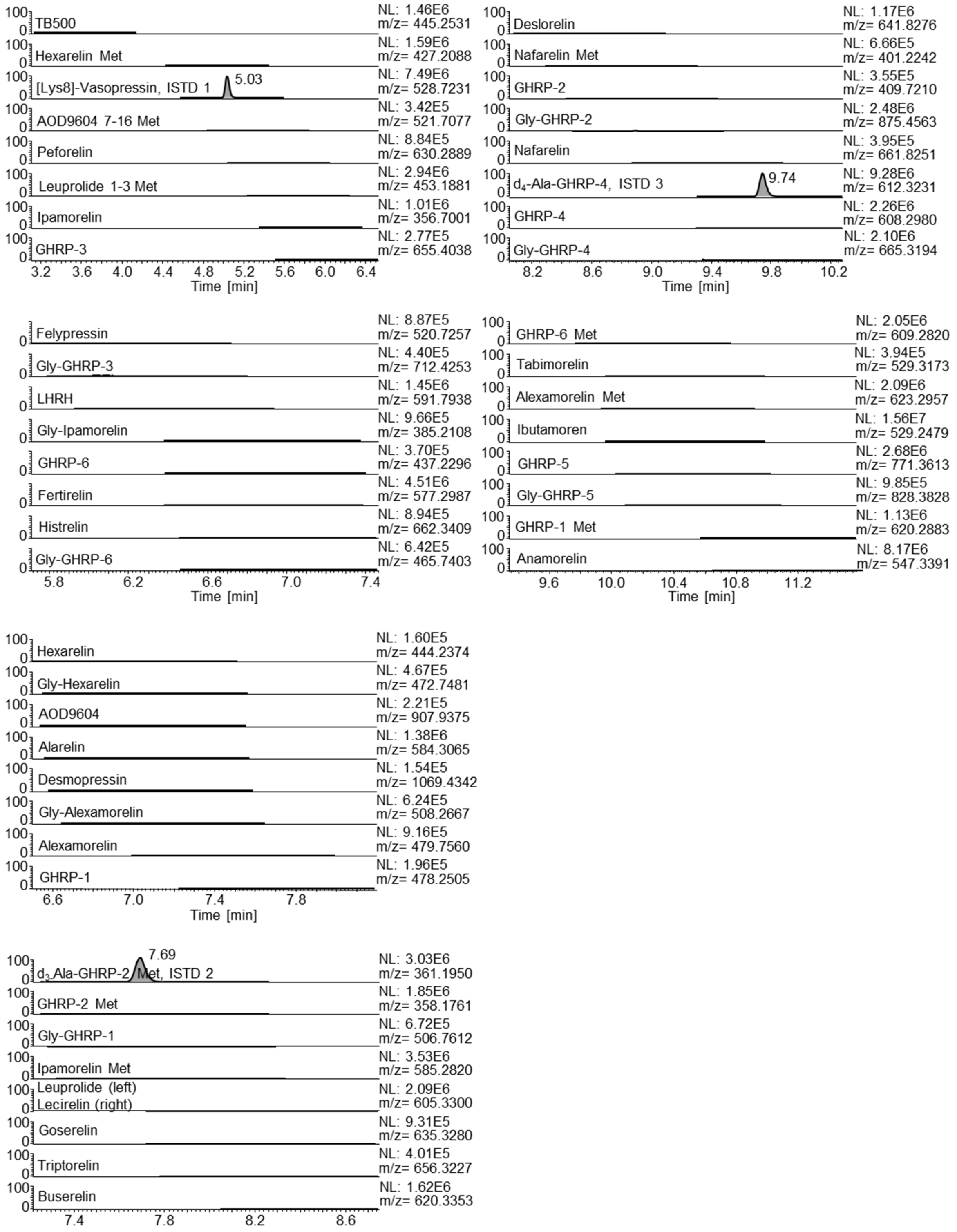

Fig. S1 Extracted ion chromatograms (mass tolerance ± 5 ppm) of a blank sample from a female volunteer obtained by a finger prick. The rows of the 3 ISTDs are also shown at their respective retention time. Normalization levels (NL) are adapted from Fig. 1

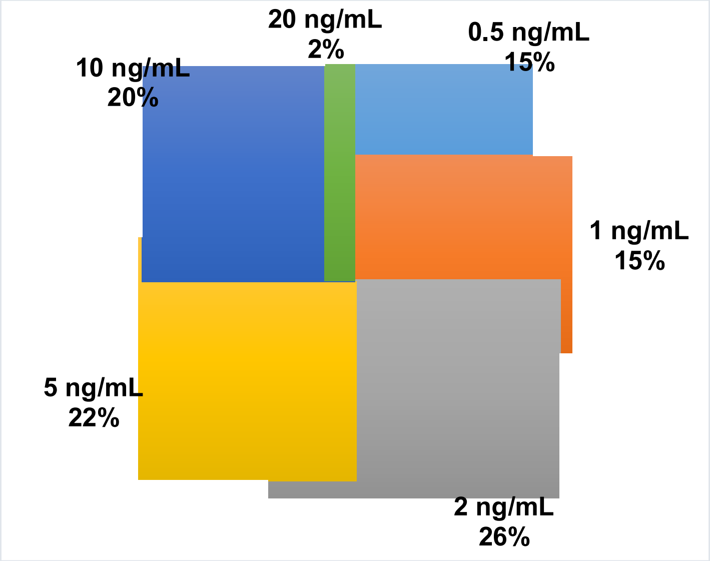

Fig. S2 The distribution of LODs. Different LODs of the analytes in the range between 0.5-20 ng/mL were obtained after analysis of a DBS sample

Supplementary tables

Table S1 Supplier, location, specified peptide purity and peptide content of the standard substances

Compound	Supplier	Location	Peptide purity [%]	Peptide content [%]
Alarelin	Auspep	Tullamarine, Australia	98	77
Alexamorelin	BMFZ	Düsseldorf, Germany	> 90*	n.a.
Alexamorelin (3-6) met.	Centic Biotec	Heidelberg, Germany	> 90	n.a.
Anamorelin	Auspep	Tullamarine, Australia	> 95	n.a.
AOD9604	Auspep	Tullamarine, Australia	98	94
AOD9604 (7-16) met.	Auspep	Tullamarine, Australia	95	73
Buserelin	Bachem	Bubendorf, Switzerland	> 95	n.a.
Deslorelin	Prospec	Rehovot, Israel	> 99	n.a.
Desmopressin	Sigma Aldrich	St. Louis, MO, USA	98	87
Felypressin	Sigma Aldrich	St. Louis, MO, USA	99.4	n.a.
Fertirelin	Bachem	Bubendorf, Switzerland	99.3	87.3
GHRP-1	BMFZ	Düsseldorf, Germany	> 90*	n.a.
GHRP-1 (3-6) met.	Centic Biotec	Heidelberg, Germany	> 90	n.a.
GHRP-2	Bachem	Bubendorf, Switzerland	97.1	75.7
GHRP-2 (1-3) met.	In-house synthesis	Cologne, Germany	99	n.a.
(d_3)-GHRP-2 (1-3) met. (ISTD 1)	In-house synthesis	Cologne, Germany	72	n.a.
GHRP-3	Pepscan	Lelystad, Netherlands	96.9	n.a.
GHRP-4	Auspep	Tullamarine, Australia	99	76
(d_4)-GHRP-4 (ISTD 2)	BMFZ	Düsseldorf, Germany	> 90*	n.a.
GHRP-5	Auspep	Tullamarine, Australia	99	74
GHRP-6	Auspep	Tullamarine, Australia	99	71.5
GHRP-6 (2-5) met.	Auspep	Tullamarine, Australia	99	76.2
Gly-Alexamorelin	Genscript	Leiden, Netherlands	98	n.a.
Gly-GHRP-1	Genscript	Leiden, Netherlands	99.4	n.a.
Gly-GHRP-2	Genscript	Leiden, Netherlands	98.4	n.a.
Gly-GHRP-3	Genscript	Leiden, Netherlands	96.2	n.a.
Gly-GHRP-4	Genscript	Leiden, Netherlands	99.3	n.a.
Gly-GHRP-5	Genscript	Leiden, Netherlands	97.7	n.a.
Gly-GHRP-6	Genscript	Leiden, Netherlands	99.1	n.a.
Gly-Hexarelin	Genscript	Leiden, Netherlands	98.1	n.a.
Gly-Ipamorelin	Genscript	Leiden, Netherlands	97.2	n.a.
Goserelin	Prospec	Rehovot, Israel	> 98	n.a.
Hexarelin	Auspep	Tullamarine, Australia	99	69.8
Hexarelin (1-3) met.	Auspep	Tullamarine, Australia	97	62
Histrelin	Sigma Aldrich	St. Louis, MO, USA	97	74
Ibutamoren	MedChem Express	Princeton, NJ, USA	> 98	n.a.
Ipamorelin	Auspep	Tullamarine, Australia	99	60
Ipamorelin (1-4) met.	Auspep	Tullamarine, Australia	99	67
Lecirelin (Dalmarelin)	Auspep	Tullamarine, Australia	97	76

Leuprolide	Sigma Aldrich	St. Louis, MO, USA	> 98	n.a.
Leuprolide (1-3) met.	Auspep	Tullamarine, Australia	95	n.a.
LHRH	Sanofi	Paris, France	GMP grade**	n.a.
[Lys8]-Vasopressin (ISTD 3)	Sigma Aldrich	St. Louis, MO, USA	98	87
Nafarelin	Sigma Aldrich	St. Louis, MO, USA	97	n.a.
Nafarelin (5-10) met.	Auspep	Tullamarine, Australia	96	71
Peforelin	Bachem	Bubendorf, Switzerland	98.2	75.3
Tabimorelin	TRC	North York, ON, Canada	98	n.a.
TB500	Centic Biotec	Heidelberg, Germany	96.1	n.a.
Triptorelin	Sigma Aldrich	St. Louis, MO, USA	> 98.8	86.2

* Peptides were obtained by custom synthesis. ‘High’ purity was estimated to be at least 90%.

** LHRH was purchased under the trade name Kryptocur® (Sanofi). ‘Highest’ purity due to GMP/clinical grade is guaranteed.

Table S2 In order to estimate the linearity of the analytes between LOD - 100 ng/mL, a linear (1st order) regression was assumed. LOD, coefficient of correlation (r), intercept, and slope are indicated

Compound	LOD [ng/mL]	Coefficient of correlation (r)	Intercept	Slope
Alarelin	5	0.9991	-0.0484062	0.0189082
Alexamorelin	1	0.9967	-0.015622	0.0046915
Alexamorelin (3-6) met.	10	0.9979	-0.0224924	0.00297027
Anamorelin	0.5	0.9998	0.00360475	0.0207482
AOD9604	10	0.9907	-0.013225	0.00170851
AOD9604 (7-16) met.	5	0.9888	-0.000170072	0.0000336746
Buserelin	5	0.9970	-0.0200733	0.00494489
Deslorelin	5	0.9952	-0.0199836	0.00326135
Desmopressin	10	0.9935	-0.0130102	0.00200175
Felypressin	0.5	0.9990	-0.00437096	0.00276108
Fertirelin	1	0.9993	-0.136574	0.0603397
GHRP-1	10	0.9919	-0.00481472	0.00121262
GHRP-1 (3-6) met.	5	0.9991	-0.00231903	0.00135471
GHRP-2	2	0.9991	-0.00763612	0.00250229
GHRP-2 (1-3) met.	2	0.9998	-0.00965567	0.00824783
GHRP-3	5	0.9973	-0.00330112	0.000470546
GHRP-4	2	0.9996	-0.0220946	0.00877921
GHRP-5	2	0.9964	-0.0447691	0.00962491
GHRP-6	2	0.9975	-0.00454555	0.000100606
GHRP-6 (2-5) met.	2	0.9982	-0.00870166	0.00292029
Gly-Alexamorelin	5	0.9990	-0.0130556	0.00222534
Gly-GHRP-1	2	0.9949	-0.00357233	0.000713673
Gly-GHRP-2	10	0.9988	-0.00776704	0.00213614
Gly-GHRP-3	5	0.9965	0.000477426	0.000186978
Gly-GHRP-4	1	0.9993	-0.0231385	0.00929519
Gly-GHRP-5	5	0.9972	-0.0352012	0.00483793
Gly-GHRP-6	2	0.9987	-0.00650108	0.00323691
Gly-Hexarelin	2	0.9986	-0.00738083	0.00263126
Gly-Ipamorelin	1	0.9984	-0.0020315	0.00106991
Goserelin	2	0.9965	-0.00252871	0.00333681
Hexarelin	10	0.9999	-0.0104049	0.00181617
Hexarelin (1-3) met.	0.5	0.9958	0.0012266	0.000346481
Histrelin	5	0.9980	-0.00350599	0.00274993
Ibutamoren	1	0.9999	0.0176974	0.0223254
Ipamorelin	0.5	0.9989	0.0016851	0.00127155
Ipamorelin (1-4) met.	0.5	0.9966	-0.00678853	0.00952589
Lecirelin (Dalmarelin)	0.5	0.9992	-0.0190056	0.00647131
Leuprolide	0.5	0.9985	-0.0173169	0.00707388
Leuprolide (1-3) met.	1	0.9981	-0.000376364	0.000287541
LHRH	2	0.9992	-0.0067248	0.00254222
Nafarelin	10	0.9862	0.00260311	0.000837598

Nafarelin (5-10) met.	2	0.9948	-0.0095818	0.00245437
Peforelin	20	0.9985	-0.0117727	0.00110986
Tabimorelin	10	0.9991	-0.00251807	0.00200117
TB500	1	0.9996	-0.000370692	0.00237711
Triptorelin	10	0.9961	-0.0113033	0.000969089
